# Effect of Applying a Real-Time Medical Record Input Assistance System With Voice Artificial Intelligence on Triage Task Performance in the Emergency Department: Prospective Interventional Study

**DOI:** 10.2196/39892

**Published:** 2022-08-31

**Authors:** Ara Cho, In Kyung Min, Seungkyun Hong, Hyun Soo Chung, Hyun Sim Lee, Ji Hoon Kim

**Affiliations:** 1 Department of Emergency Medicine, Yonsei University College of Medicine Seoul Republic of Korea; 2 Department of Research Affairs Biostatistics Collaboration Unit Yonsei University College Seoul Republic of Korea; 3 CONNECT-AI Research Center Yonsei University College of Medicine Seoul Republic of Korea; 4 Department of Emergency Nursing Yonsei University Health System Seoul Republic of Korea

**Keywords:** voice recognition, artificial intelligence, natural language processing, emergency department, triage

## Abstract

**Background:**

Natural language processing has been established as an important tool when using unstructured text data; however, most studies in the medical field have been limited to a retrospective analysis of text entered manually by humans. Little research has focused on applying natural language processing to the conversion of raw voice data generated in the clinical field into text using speech-to-text algorithms.

**Objective:**

In this study, we investigated the promptness and reliability of a real-time medical record input assistance system with voice artificial intelligence (RMIS-AI) and compared it to the manual method for triage tasks in the emergency department.

**Methods:**

From June 4, 2021, to September 12, 2021, RMIS-AI, using a machine learning engine trained with 1717 triage cases over 6 months, was prospectively applied in clinical practice in a triage unit. We analyzed a total of 1063 triage tasks performed by 19 triage nurses who agreed to participate. The primary outcome was the time for participants to perform the triage task.

**Results:**

The median time for participants to perform the triage task was 204 (IQR 155, 277) seconds by RMIS-AI and 231 (IQR 180, 313) seconds using manual method; this difference was statistically significant (*P*<.001). Most variables required for entry in the triage note showed a higher record completion rate by the manual method, but in the recording of additional chief concerns and past medical history, RMIS-AI showed a higher record completion rate than the manual method. Categorical variables entered by RMIS-AI showed less accuracy compared with continuous variables, such as vital signs.

**Conclusions:**

RMIS-AI improves the promptness in performing triage tasks as compared to using the manual input method. However, to make it a reliable alternative to the conventional method, technical supplementation and additional research should be pursued.

## Introduction

An essential role of a hospital emergency department (ED) is to prioritize treatment for patients according to urgency and symptom severity [1]. This role is related to the nature of ED work, where unpredictable situations often occur, and resources are limited owing to crowding [2,3]. Because emergency care demands higher efficacy to manage growing patient volumes, a prompt and evidence-based triage system is required to provide safe and optimal care [4]. Most EDs are equipped with a “triage system” that immediately classifies the severity of a patient’s symptoms in the period between patient arrival and start of clinical steps by ED physicians [5]. Initial severity classification includes checking vital signs and recording patient history by conversing with the patient or guardian [6].

Because the results derived through the triage system must be immediately recorded and shared with the medical staff in charge of the next process, a prompt triage system is crucial for an efficient ED. In addition, the results from the triage system are reported to have a significant influence on clinical outcomes [7-10]. Therefore, the accuracy of the triage process is also important for the safe operation of an ED. However, the existing triage system is mostly operated by medical staff rather than physicians, and there may be bias due to the subjective measurement [5]. In addition, because the time required in the triage unit has been prolonged because of the COVID-19 outbreak, rapid and reliable patient classification is threatened in EDs [11].

Recent advances in machine learning and natural language processing (NLP) are a prominent development in health informatics and are relevant in emergency medicine [12]. Although NLP has been established as an important tool when using unstructured text data, most studies in the medical field have been limited to a retrospective analysis of text entered manually by humans on electronic medical records [6,13-18]. There is little research that addresses applying NLP to the conversion of raw voice data generated in the clinical field into text using speech-to-text (STT) algorithms [1,19]. Therefore, the aim of this study was to investigate the promptness and reliability of a real-time record input assistance system developed with STT and NLP technology and compare it to the manual method used by triage medical staff who perform time-critical tasks in the ED.

## Methods

### Ethics Approval

This study was conducted in accordance with the revised Declaration of Helsinki and was reviewed and approved by the Institutional Review Board of Severance Hospital, South Korea (approval number 4-2020-0598).

### Study Setting and Participants

We performed a prospective interventional study. This study was conducted at a Level 1 ED at a tertiary hospital located in northwestern Seoul (the capital city of South Korea), where 90,000 patients visit annually. The hospital’s ED is responsible for receiving patients who cannot be stabilized in this catchment area. Participants were recruited through an official announcement period from November 1, 2020, to the end of January 2021. Among the nurses performing triage work in the hospital’s ED, 19 nurses who listened to the contents and process of the study voluntarily agreed to participate in the study. They had more than three years of ED work experience. Exclusion criteria included candidates who (1) withdrew their intention to participate, or (2) had physical symptoms that made it difficult for them to wear a voice recognition microphone. Informed consent was obtained from all participants before enrollment.

### Machine Learning Framework

Because conversations in the triage unit contained a large amount of information and noise, a device that can select and record these conversations was needed. A machine learning framework created by Selvas AI Inc (Seoul, Republic of Korea) was used in this study. The voice recognition solution provided by Selvas analyzes sound information and converts it into text, commands, and various forms of information. The application of continuous word recognition engines, which recognize unstructured speech, has expanded to different fields; for example, a speech recognition engine in this study has been exclusively developed for the medical field. In our ED, the triage nurses are supposed to record the results of performing a task in a triage note. This triage note consists of the following items: chief concern, past medical history, the presence of allergic diseases, vital signs such as systolic and diastolic blood pressure, heart rate, respiratory rate, body temperature, and oxygen saturation. To train the engine, triage nurses who agreed to participate in this study performed the clinical practice wearing Bluetooth microphones (Aftershokz Aeropex, AS 800, Aftershokz LLC). Voice recording files that passed through the engine were immediately converted into textual data, without prior editing, and stored as log records. Subsequently, the engine repeatedly trained the NLP to fill the items constituting the triage note using the transcribed textual data. The Bluetooth microphone was selected as a component of a noise-resistant system in accordance with the ED environment where various noises exist, and a mobile recording system was built to ensure its mobility. The Bluetooth microphones, voice recognition software, and systems using computers connected to them were installed in the triage unit, and voice data were recorded during the data collection period. For 6 months, 1717 triage cases were collected, and the machine learning engine was trained to recognize the sound using these voice data, convert it into textual data, and perform the subsequent NLP. Consistent with the current triage note format, the system was trained to classify the chief concern of each patient into 1 of 52 categories, and the past medical history was processed into 13 categories through NLP. In the triage note used in this ED, up to 3 chief concerns and the medical history can be entered. The presence of allergic diseases was configured to be treated as a binary input, and variables representing vital signs were treated as continuous variables.

For accurate voice interval detection in a noisy environment, the end-point detection module was optimized in the machine learning engine. By distinguishing various nonstationary noises through continuous adaptive learning for noise coming through the Bluetooth microphones, a deep neural network end-point detection module was developed with high accuracy in detecting energy-based voice sections of the existing method. The voice interval detection module optimized for the voice environment input to the Bluetooth microphone was advanced, and sound using the collected and processed purified voice database and converted textural data was applied for language model learning.

### Study Protocol

**Figure 1:**
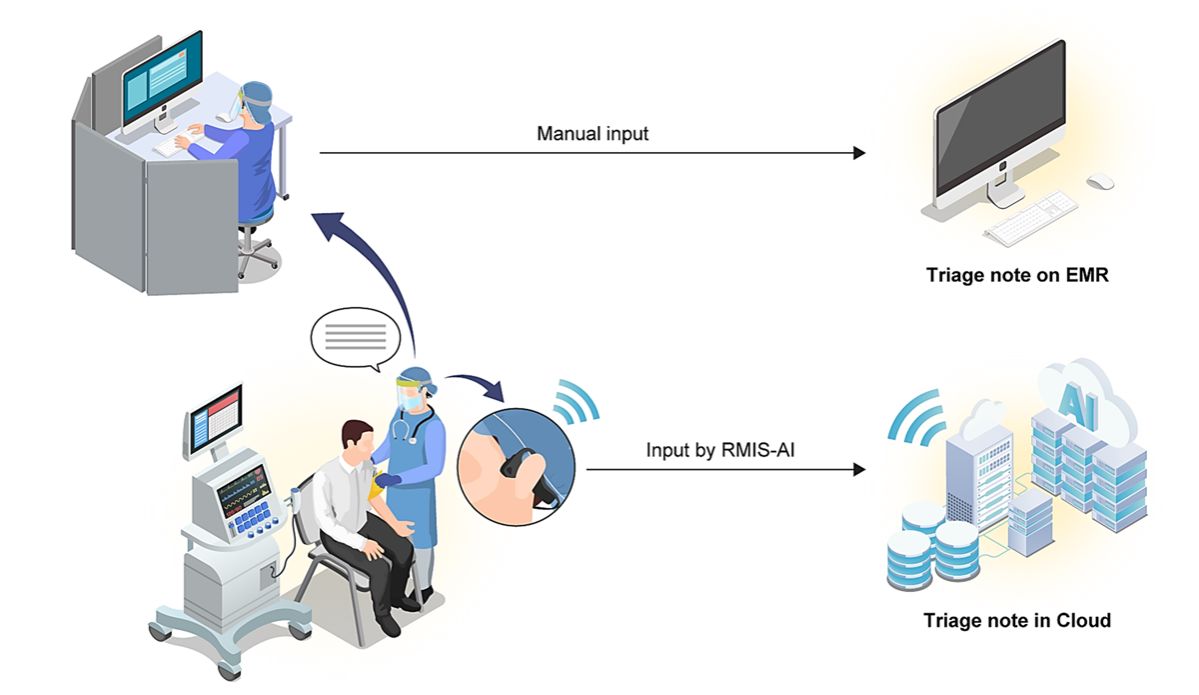
Two-input process of charting, RMIS-AI (real-time medical record input assistance system with voice artificial intelligence) vs manual input. EMR: electronic medical record.

From June 4, 2021, to September 12, 2021, a real-time medical record input assistance system with voice artificial intelligence (RMIS-AI) built using a trained engine was prospectively applied to the clinical practice in the triage unit where the patients meet the medical staff for the first time. RMIS-AI is a tool that assists in recording triage notes through voices. In other words, it secures the mobility of a triage nurse by replacing the record input means with voice instead of the desktop computer keyboard. RMIS-AI was implemented on a cloud-based network separate from the hospital electronic medical record (EMR) system. During the study period, participants wearing Bluetooth microphones recorded triage data in the EMR by asking detailed questions to each patient and checked vital signs. Simultaneously, they also recorded the data through RMIS-AI in the same format using their voice. Because the participants used a closed-loop communication method that reconfirmed the meaning of the patient’s words and uttered them, the information obtained from the patients could be delivered by the participant’s voice rather than the patient’s voice. The input process of charting through RMIS-AI was blind to the nurses, and they monitored the EMR input process as usual when performing the triage task. The contents and time of the triage log finally created in both ways were stored in the hospital EMR log and cloud storage, respectively (Figure 1). The data stored in each database were automatically extracted and used for our research.

### Outcome Measures

The primary outcome was the time for participants to perform the triage task. It was defined as the time from the patient’s arrival at the triage unit to the completion of the triage note. We measured these times using data stored in the hospital EMR for manual input and cloud storage for RMIS-AI. The secondary outcome metrics were the record completion rate and the accuracy of RMIS-AI compared to manual input by EMR.

### Statistical Analysis

The sample size was calculated from the mean time taken by performing the triage task in a conventional method for 100 cases before the intervention was started. We considered that the RMIS-AI producing a mean difference of 20 seconds with standard deviation difference of 2 seconds would be considered clinically significant (*P*<.05, statistical power=0.95). Therefore, the required sample size was calculated to be 952 cases by G-power 3.1.9.7, requiring a total of 1057 triage cases considering a 10% dropout rate. In this paper, categorical variables are presented as counts and percentage. Continuous data are presented as mean or median and SD or interquartile range. The Mann-Whitney *U* test was used to identify the differences of primary outcome between the 2 groups. Differences in record completion rates between the 2 methods were compared using the McNemar test. The result was considered statistically significant at *P*<.05. The intraclass correlation coefficient (ICC) using the 2-way mixed effects model, absolute measurement, and single measurement were used to evaluate the interrater reliability of continuous data between the 2 groups [20], and this reliability was visualized using the Bland-Altman plot. The degree of agreement for all variables was represented as a proportion. The accuracy of the chief concern and past medical history was classified into complete, partial, and fail. All statistical analyses were performed using R 3.6.0 (The R Foundation for Statistical Computing).

## Results

**Figure 2:**
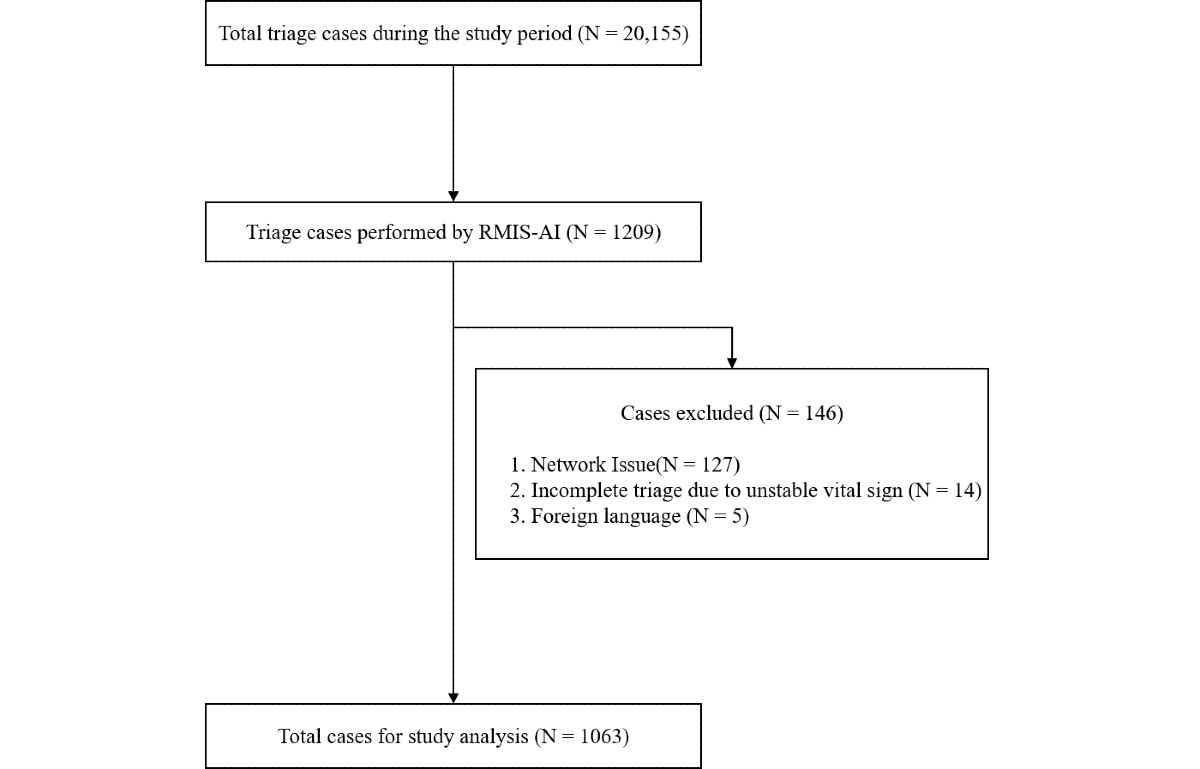
Flowchart of case inclusion. RMIS-AI: real-time medical record input assistance system with voice artificial intelligence.

During the study period, a total of 20,155 triage cases were processed at the hospital’s ED, at an average of 194 cases per day. Among them, 1209 (6%) triage tasks were performed by the participants. After 146 cases were excluded by the criteria shown in Figure 2, a total of 1063 cases were used for study analysis.

**Figure 3:**
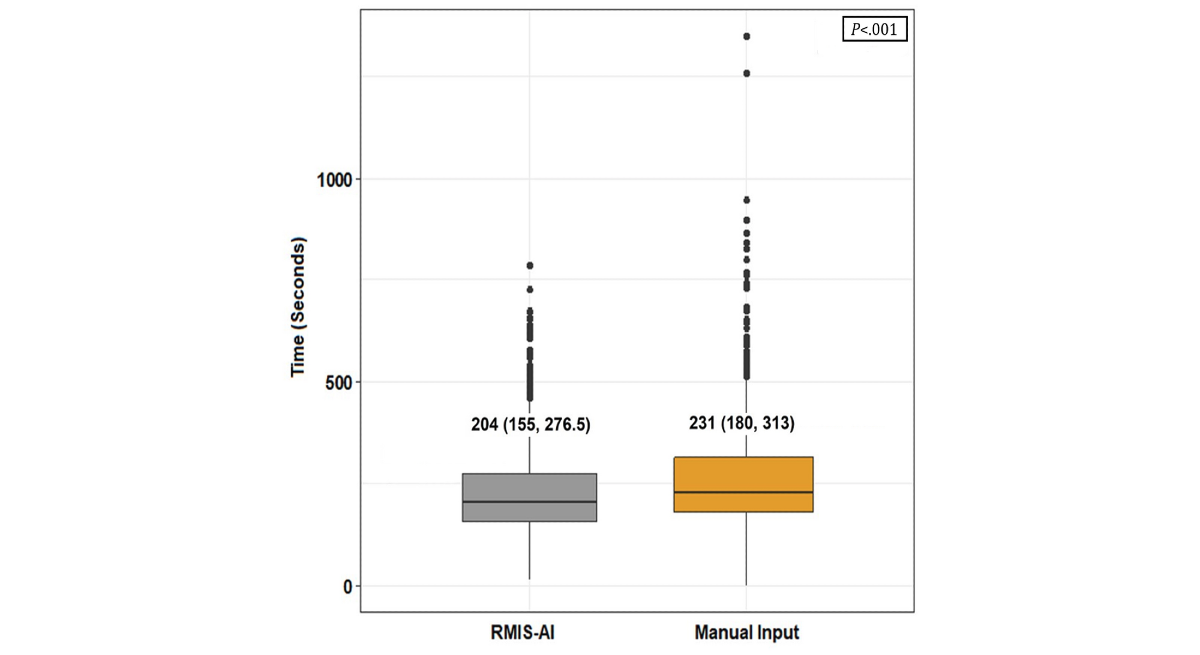
Comparison of median time for triage task, RMIS-AI (real-time medical record input assistance system with voice artificial intelligence) vs manual input.

The median time for participants to perform the triage task was 204 (IQR 155, 277) seconds with RMIS-AI and 231 (IQR 180, 313) seconds using manual input by EMR. The difference between the 2 methods was statistically significant (*P*<.001), as shown in the box plot in Figure 3.

The record completion rates of both methods for all triage cases are shown in Table 1. In the triage notes recorded by RMIS-AI, the first chief concern showed the highest record completion rate (81.84%), and all variables of vital signs that should be recorded as continuous variables showed comparable record completion rates of over 50% except for the respiratory rate. In most variables of the triage note, RMIS-AI showed a lower record completion rate than the manual method. However, in terms of recording additional chief concerns and past medical history, RMIS-AI showed a higher record completion rate than the manual method, which was statistically significant.

The accuracy of reproducing records by RMIS-AI for all variables is summarized in Table 2. In this study, only systolic blood pressure, diastolic blood pressure, oxygen saturation, and chief concern represented an accuracy of more than 50%, implying that RMIS-AI reproduced these variables recorded by the manual method by more than 50%. Furthermore, categorical variables such as past medical history and history of allergic episodes entered by RMIS-AI showed less accuracy than other variables.

**Figure 4:**
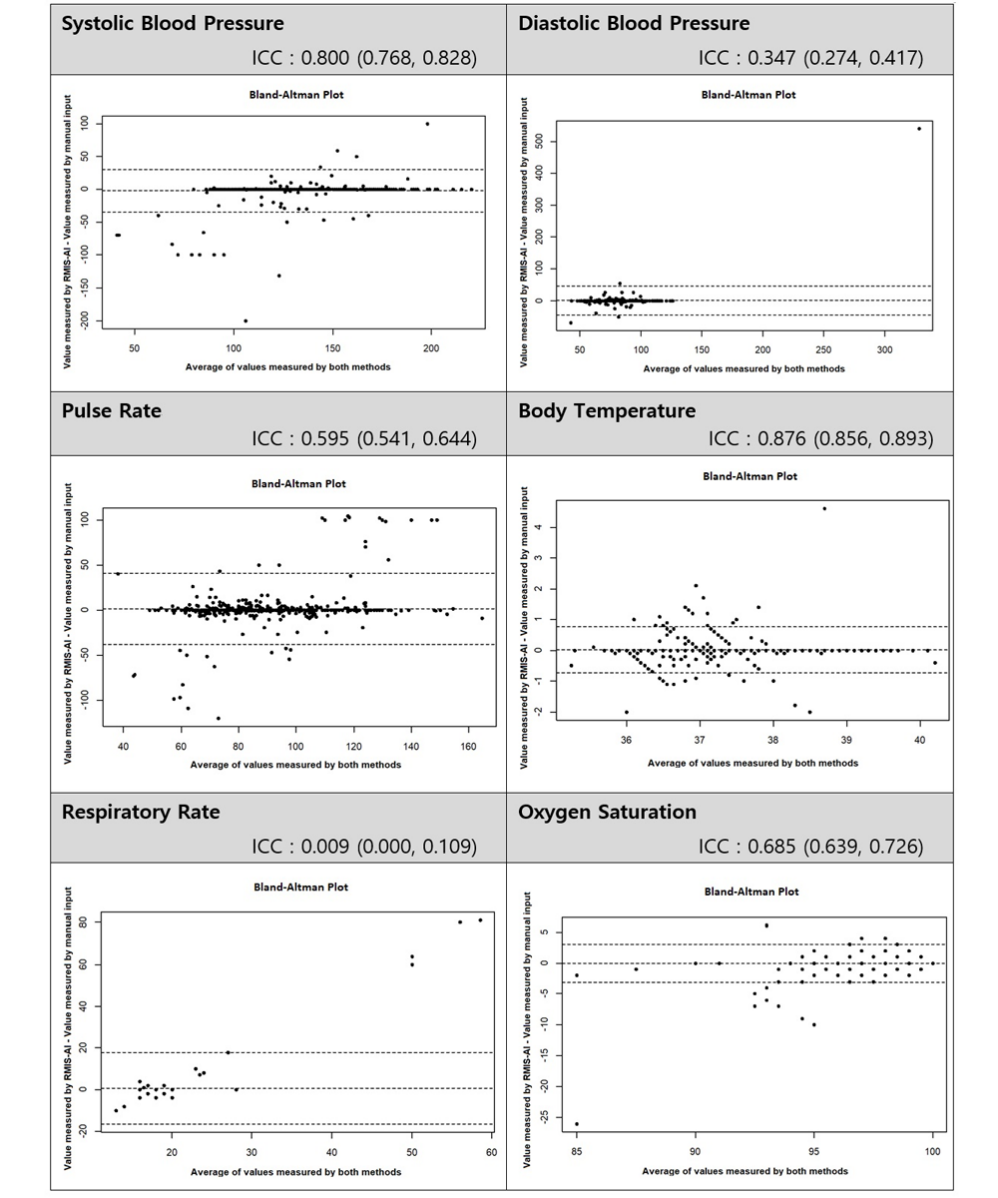
Interrater reliability for continuous variables between 2 methods. ICC: intraclass correlation coefficient; RMIS-AI: real-time medical record input assistance system with voice artificial intelligence.

Figure 4 shows the interrater reliability for continuous variables between the 2 methods. The ICC of systolic blood pressure and body temperature were 0.800 and 0.876, respectively, indicating substantial interrater reliability.

**Table 1:** Record completion rates of both methods.

Variable	Record completion cases, n (%)		*P* value
	RMIS-AI^a^	Manual input	
Chief concern, 1st	870 (81.84)	1063 (100)	<.001
Chief concern, 2nd	515 (48.45)	397 (37.35)	<.001
Chief concern, 3rd	230 (21.64)	106 (9.97)	<.001
History of allergic episode	257 (24.18)	1063 (100)	<.001
Past medical history, 1st	383 (36.03)	1030 (96.90)	<.001
Past medical history, 2nd	127 (11.95)	32 (3.01)	<.001
Past medical history, 3rd	27 (2.54)	12 (1.13)	.02
Systolic blood pressure	580 (54.56)	923 (86.83)	<.001
Diastolic blood pressure	578 (54.37)	923 (86.83)	<.001
Pulse rate	613 (57.67)	925 (87.02)	<.001
Respiratory rate	382 (35.94)	923 (86.83)	<.001
Body temperature	607 (57.10)	1061 (99.81)	<.001
Oxygen saturation	584 (54.94)	926 (87.11)	<.001

^a^RMIS-AI, real-time medical record input assistance system with voice artificial intelligence.

**Table 2:** Accuracy of RMIS-AI^a^ compared to the manual method.

Variable			Cases with reproduction and cases with records by manual method, n/N (%)
**Chief** **concern**			
	Complete reproduction^b^	366/1063 (34.43)	
	Partial reproduction^c^	190/1063 (17.87)	
	Failed reproduction^d^	507/1063 (49.41)	
**Past medical history**			
	Complete reproduction	226/1030 (21.94)	
	Partial reproduction	5/1030 (0.49)	
	Failed to reproduction	799/1080 (73.98)	
History of allergic episode			158/1063 (14.68)
Systolic blood pressure			516/923 (55.90)
Diastolic blood pressure			495/923 (53.63)
Pulse rate			352/925 (38.05)
Respiratory rate			340/923 (36.84)
Body temperature			484/1061 (45.62)
Oxygen saturation			465/926 (50.22)

^a^RMIS-AI: real-time medical record input assistance system with voice artificial intelligence.

^b^All the values by manual input were reproduced by RMIS-AI.

^c^Partial values by manual input were reproduced by RMIS-AI.

^d^No values by manual input were reproduced by RMIS-AI.

## Discussion

### Principal Findings

Previous study results have proven that prolonged waiting times and crowding are factors that reduce patient satisfaction and impair safety in the ED [21-25]. Long waiting times are known as the main cause of leaving without being seen after enrollment [26]. Leaving without being seen is considered an indicator of timeliness and effectiveness, which falls within the quality of care, as defined by the US Institute of Medicine, and poses a safety threat because it limits the options for patients to seek treatment elsewhere [27,28]. This study’s results confirmed that the use of RMIS-AI in the ED shortens the time to perform the triage task. In cases where the patient influx of ED is rapidly increasing, reducing the time taken to perform the triage task could contribute to reducing patient waiting time. The triage task mainly includes taking patient history through conversations and measuring vital signs. Typically, these actions and the recording of patient information are performed in separate steps. It is estimated that the RMIS-AI developed using voice recognition technology in our study reduced the time to perform the triage task by combining these tasks in a single step. After the COVID-19 outbreak, the screening process for patients visiting ED has been strengthened, resulting in a longer delay during the input phase [11]. In addition, medical staff who face the ED patients for the first time wear personal protective equipment to protect them from potential risk of infection [29], which made multitasking difficult for the medical staff of the triage unit. Therefore, the RMIS-AI developed using AI technology has proven its potential as a supportive solution to improve the quality of clinical practice in response to the new digital era as well as after the COVID-19 pandemic in the ED.

The record completion rates of RMIS-AI were inferior to the manual input by EMR in our study, especially in the input of allergy history or past medical history. In the case of categorical variables, such as allergy history or past medical history, NLP is more difficult than in the case of continuous variables, such as systolic blood pressure and pulse rate, because it is expressed in a wide variety of phrases rather than simple utterances. Korean is an agglutinative language and one of the morphologically rich and typologically diverse languages. Auxiliary, adverbial case markers, word spacing inconsistency, and the variety of expressions of predicates with the same meaning make NLP using Korean difficult. [30]. The engine was trained to input categorical variables, such as chief concern into 1 of 52 categories, and 13 categories for past medical history and binary format for allergy history. Because the sensitivity of the engine increases as there are more categories that can be input through NLP of the transcribed textual data, it was estimated that the record completion rate is low for past medical history and allergy history with relatively few categories. In addition, inferior results compared to manual input are observed presumably because triage nurses could not monitor the recording by RMIS-AI during triage tasks, and only recording by EMR was possible as usual. If the recording system of triage tasks using RMIS-AI compensates the conventional method, despite the time for triage task being longer than that reported in the study, it is expected that the record completion rate will be comparable to that of the manual method. For example, if triage nurses find that variables to be input by voice recognition have not been recorded during the task, they can speak to compensate for the missing variables. While recording patient history, it is common for triage performers to omit inessential information intentionally or forget acquired information. It has been reported that errors due to the inexperience of triage performers may adversely affect patients [4,5]. The recording by RMIS-AI involves relatively little subjectivity from the performer. Thus, RMIS-AI represents an alternative method that can offset the negative effects that occur because of the subjectivity of the triage performer. By recognizing various input values while recording patient history, it is possible to capture more detailed information that could not be detected using the conventional method. In this context, in the case of variables with multiple inputs, such as chief concern and past medical history, the record completion rate for subitems input by RMIS-AI was superior to that of the manual input.

In our study, it is assumed that the difference between the variables with and without relatively favorable accuracy is due to the complexity of NLP. NLP is still being developed as an artificial intelligence field, and because there is no standardized format, its performance is different depending on the type and amount of training data as well as the deep learning method applied [31,32]. For variables such as vital signs, the process ends with the triage nurse’s voice passing through STT and charting the converted text numerically, but categorical variables such as chief concern should be categorized as textual data converted by STT into 1 of 52 categories through NLP. Variables of past medical history and history of allergic episode that the system was trained to classify into fewer categories had a lower record completion rate and failures for accurate production compared with the variables of chief concerns; this result is also presumed to be caused by the differences in NLP. In addition, the low record completion rate of RMIS-AI also led to inferior accuracy not being able to reproduce triage notes. In particular, the inferior accuracy of numerical variables applying the relatively uncomplicated NLP was attributed to the low record completion rate.

The reliability of pulse rate was lower than that of other vital sign values because there was a time difference between the input through RMIS-AI and the manual input because triage nurses record the pulse rate by watching the monitoring being measured as a continuous waveform. This result can be explained by the Bland-Altman plot, where the error range in the input value is narrow. In addition, the low ICC value of the respiratory rate was due to the less amount of data and low variability.

NLP is a tool that can structure unstructured textual data and enable the use of unstructured voice data that historically have not been used in the medical field. Previous studies have reported that the predictive performance of clinical outcomes is improved when unstructured textual data are used for machine learning in the medical field [13,15,16,33]. However, for unstructured textual data to be used in actual clinical practice rather than only in retrospective analyses, an environment in which STT is performed in real time should be developed. Most previous studies using textual data performed a retrospective analysis of text recorded on EMR using machine learning; therefore, they were not sufficient evidence in terms of improving clinical practice in the ED, which is a time-critical setting. Our study did not focus on prediction using a machine learning model with NLP but investigated the potential application of performing STT in a real clinical field through a prospective design. In this study, the machine learning framework was trained on unprocessed audio data. This approach can lead to an easy transition to a new system for acute clinical settings where decision-making should be efficient and precise in the digital era [19].

This study has several limitations. Although our study was conducted in a prospective design, a study using a randomized controlled design is needed to obtain definitive evidence that the RMIS-AI can replace the conventional method. Second, the completeness and accuracy of the triage note by the current RMIS-AI are insufficient to safely replace the conventional manual input method. If NLP for the recording of triage note recording is learned using additional training material, it can be improved**.** Third, the reduction in time taken to perform the triage task does not guarantee improvements in patient outcomes. Therefore, the relationship between the use of RMIS-AI and improvement in clinical outcomes on patients in the ED should be investigated. Finally, the study was performed at an ED in a single tertiary hospital; thus, there is a limit to generalizing the research results.

### Conclusions

In this study, we confirmed that the promptness in performing triage tasks improved using RMIS-AI developed with STT and NLP technology compared with the manual input method, but technical supplementation was required to deal with the current level of inferiority in sensitivity and accuracy. If similar studies are conducted to confirm the potential of such technologies in clinical practice, artificial intelligence could evolve as a supportive tool to improve patient experience.

## Abbreviations

ED: emergency department
EMR: electronic medical record
ICC: intraclass correlation coefficient
NLP: natural language processing
RMIS-AI: real-time medical record input assistance system with voice artificial intelligence
STT: speech-to-text
